# NORMAL HUMAN VARIATION: REFOCUSSING THE ENHANCEMENT DEBATE

**DOI:** 10.1111/bioe.12045

**Published:** 2013-08-02

**Authors:** Guy Kahane, Julian Savulescu

**Keywords:** enhancement, normal variation, status quo bias, moral psychology

## Abstract

This article draws attention to several common mistakes in thinking about biomedical enhancement, mistakes that are made even by some supporters of enhancement. We illustrate these mistakes by examining objections that John Harris has recently raised against the use of pharmacological interventions to directly modulate moral decision-making. We then apply these lessons to other influential figures in the debate about enhancement. One upshot of our argument is that many considerations presented as powerful objections to enhancement are really strong considerations in favour of biomedical enhancement, just in a different direction. Another upshot is that it is unfortunate that much of the current debate focuses on interventions that will radically transform normal human capacities. Such interventions are unlikely to be available in the near future, and may not even be feasible. But our argument shows that the enhancement project can still have a radical impact on human life even if biomedical enhancement operated entirely within the normal human range.

The debate about human enhancement rages on. In this paper, we shall diagnose some common mistakes in thinking about the ethics of human enhancement. These mistakes affect the thinking not only of critics of biomedical enhancement, but also of some of its prominent defenders.

One source of these errors is the common focus, in the debate about biomedical enhancement, on the prospect of interventions that would radically transform (even transcend) normal human capacities – much of the debate has focused, for example, on drugs that might dramatically increase intelligence to super-human levels, or treatments that would extend our life span indefinitely. It is not by accident that books criticizing enhancement have grand titles such as *Our Posthuman Future*, *The Future of Human Nature*, and *The Case Against Perfection*.^1^

It is unlikely, however, that such radical interventions will be available in the near future, if they are even feasible. And this focus on what we shall call *supranormal enhancement* can be an obstacle to clear thinking about the forms of biomedical enhancement that are almost certainly feasible, and in fact likely to be available soon – the forms of enhancement that operate *within* the existing range of human capacities and dispositions.^2^ We call this *normal range human enhancement*. It is common to ignore or dismiss these less dramatic forms of enhancement. But we shall argue that this is a serious mistake.

The focus on extreme forms of biomedical enhancement encourages several related mistakes. The first mistake is to think of biomedical enhancement as primarily (even necessarily) an external intervention that introduces some radical new element to our mental life, and can thus distort our natural mental apparatus. But we shall see that this *isn't* how biomedical enhancement usually works, and not what it usually works on. Biomedical enhancements typically modulate naturally existing substances and processes. The second mistake is to forget that the current levels of these substances and processes are set by largely blind processes, meaning that it's highly implausible that they are already at an optimal level, relative to our values. The third mistake is to forget that considerable individual differences within the normal human population mean that, even if there *was* a natural optimal level, the natural state of very many people is far from optimal.

One implication of our argument is that the project of enhancement can still have a radical impact on human life even if biomedical enhancement operated *entirely within the normal human range*. Another upshot is that many considerations that are presented as powerful, even decisive, objections to biomedical enhancement aren't objections at all – they are really strong considerations in *favour* of biomedical enhancement, *just in a different direction*.

In order to illustrate these mistakes, we shall largely focus on an objection to biomedical moral enhancement that has been pressed by John Harris in several recent papers. We shall identify a number of problems with this objection. But our main aim isn't to criticize Harris, or defend moral enhancement. It is rather to show that the mistakes we diagnose are so pervasive that they can even bias the thinking of those, like Harris, who usually defend human enhancement. These mistakes are an obstacle to progress in the human enhancement debate.

## Harris on Serotonin

John Harris has been a staunch advocate of biomedical human enhancement for a long time, but in recent years he has expressed strong skepticism about the idea that biomedical interventions could (and should) be used to directly influence our *moral* capacities.^3^

In a series of papers, some co-written with Sarah Chan, Harris offers several arguments against the use of biomedical means to influence moral decision-making. Harris is skeptical, or worse, about the idea that pharmacological and other biomedical interventions can be used to improve moral behavior. For example, Harris and Chan think that:

The most reliable moral enhancement technologies that we have are education, parental and peer influence, and example and moral reasoning. We would welcome smart pills (or perhaps good pills) that could improve on these technologies, but they seem as far off as ever.^4^

One recurring set of objections relates to a recent study reporting the effects on moral decision-making of the selective serotonin re-uptake inhibitor Citalopram, a pill that *already* exists, and indeed is already widely used as an antidepressant.^5^ Subjects who were given Citalopram, and who thus had elevated levels of the neurotransmitter serotonin, were less likely to endorse utilitarian solutions to moral dilemmas that require directly killing or harming someone in order to save a greater number, such as the notorious Footbridge case which asks whether we should push an innocent person onto the path of a runaway train in order to save the lives of five other innocent individuals. As the researchers interpret this finding, the elevated levels of serotonin led to an increase in subjects' aversion to directly harming others, and thus to a decrease in their willingness to endorse utilitarian answers to moral questions.

Harris and Chan aren't impressed by this result. They write that ‘… if serotonin affects moral behaviour, it does so adversely by impairing moral judgment, subjugating it to emotional instinct.’^6^ Elsewhere they describe serotonin as a ‘moral de-enhancer’.^7^ Harris and Chan offer several grounds for these claims. We shall consider these in turn, and then draw more general lessons for the debate about enhancement.

## Disposing People Against Utilitarian Responses

The first ground is that increased serotonin disposes people against utilitarian answers to moral questions.

It's worth pointing out first that as an interpretation of the results of this study, this claim is somewhat inaccurate. First, the evidence so far only shows that higher levels of serotonin dispose against utilitarian answers to a very specific kind of moral dilemma, involving killing or seriously harming people in order to save a greater number. There is no evidence, and it is highly unlikely, that increased serotonin makes people generally less utilitarian across the board.^8^ Second, even if we consider only this particular type of moral context, serotonin disposes people to make judgments that are less utilitarian only if by utilitarianism we mean a very simple version of Act Utilitarianism, understood not only as a criterion of rightness but also as an explicit decision procedure. But many forms of utilitarianism recommend having dispositions that lead to better consequences overall, even if they lead to sub-optimal results in unusual contexts. Aversion to seriously physically harming other people is a classic example of such a disposition. Even if it pushing a fat man to death to save five others leads to better consequences in *this* particular context, having a disposition that makes it hard to harm another human being in this way might have far better consequences overall.

Even if we set aside these points, and accept the above claim, it would of course show that serotonin is a de-enhancer only if we already endorse utilitarianism. Needless to say, it is a substantive question whether some biomedical intervention counts as a moral enhancer or de-enhancer – utilitarians and deontologists will unsurprisingly often disagree.

But we can also set aside this familiar ethical debate. What is important for our purposes is that if raising serotonin is a moral de-enhancer, this *isn't* an argument against using biomedical means to influence moral decision-making. It is an argument against *raising* serotonin. This means that, on its own, it is rather a straightforward argument for seeing interventions that *lower* serotonin as *good* examples of biomedical enhancers. In fact, there exist a number of simple and effective interventions, such as tryptophan depletion, that effectively lower serotonin, and which have been studied extensively in the laboratory.^9^ Although the effect of these interventions on responses to moral dilemmas have not yet been studied, they are likely to lead to a decrease in aversion to direct harm, and thus to an increase in utilitarian judgment in trolley-like situations.

Moreover, if the complaint is that Citalopram makes people more deontological, and that this is a bad thing, then of course this overlooks the point that most people are *already* highly deontological in their thinking generally, and specifically in contexts involving harming one person to maximize welfare. This tendency has been demonstrated by numerous empirical studies.^10^ What Citalopram apparently does is make people *even more* deontological. So again, if our aim was to increase utilitarian moral dispositions, this again strongly suggests that we need to reduce serotonin levels, not embrace the existing state of things.

So Harris's first objection turns out to really be a strong argument *for* using biomedical means to influence moral decision-making – just in the opposite direction.^11^

## Increasing the Influence of Emotion on Moral Decision-Making

The second ground that Harris and Chan offer against serotonin as an enhancer is that increased serotonin leads to an increase in the influence of emotion on moral decision-making. This interpretation is also suggested by the quote above about ‘subjugating’ moral decision-making to ‘emotional instinct’.

Notice that this is really a separate objection to the previous one. Some emotions might make us more deontological, but others could make our responses more utilitarian.^12^ If the problem was that Citalopram makes us less utilitarian, then it matters little whether it does so through emotion or in some other way. If the problem is with the influence of emotion, then this relates to a general worry about the biasing influence of emotion on morality, a worry that even some deontologists might share.

You might think that at least *this* interpretation of the worry can support a strong objection to pharmacological interventions that influence moral decision-making by modulating emotion. But this objection also doesn't work.

Again, if increased serotonin leads to increased emotional influence on our moral deliberation, and this is a bad thing, then it again seems to follow that we should reduce serotonin levels, and thereby decrease this biasing influence. Again, we get not an argument against biomedical interventions in moral decision-making, but a strong argument in their favour, just in the opposite direction.

And again, this argument can be generalized. Suppose you thought that emotion generally biases moral decision-making, or somehow reduces freedom, and that the ideal form of moral deliberation should be based on pure reason.^13^ This is an incredibly controversial picture of morality, both empirically and philosophically, but we can set this point aside for our purposes.^14^ The main point is, again, that everyone is *already* influenced by serotonin, and thus by emotion, even without taking Citalopram. And there is also a vast amount of evidence about the influence of emotion on moral decision-making in very many moral contexts.^15^ So if we adopt this picture of ideal moral deliberation, then it turns out that we are all already, in varying degrees, in a profoundly ‘corrupt’ and unfree state.^16^ And this seems to mandate a truly radical project of biomedical enhancement that would aim to remove, or at least mimimize, the influence of emotion on moral deliberation. So again this argument backfires.^17^

In this way, even those who reject the ambitious positive project of trying to *perfect* human virtue seem at least committed to the *negative* project of aiming to *minimize* the influence of biasing factors on our moral psychology. But this negative project is also ambitious, and very likely to require extensive biomedical intervention.

There is a general lesson here about criticism of biomedical enhancement. For pretty much every objection to biomedical enhancement, it is possible to reply: ‘If what worries you about enhancement is X, then why shouldn't we try to *enhance X*?’ For example, if your worry about human enhancement is that it would threaten our openness to the unbidden, or our solidarity with others,^18^ or our autonomy,^19^ then how can you object to biomedical interventions aimed to *increase* people's openness to the unbidden, or their autonomous capacities? Until we are given an explanation of why *this* kind of enhancement is problematic (and it couldn't be problematic because it threatens, say, openness to the unbidden), it is hard to take these worries as presenting any kind of *general* argument against biomedical enhancement.

## The Bad Effects of Increasing the Aversion to Directly Harming Others

The third ground offered for the claim that serotonin is a ‘moral de-enhancer’ is that increasing the aversion to harming others will have bad effects.

Harris and Chan offer the example of Jasper Schuringa, hero of Flight 253, a plane passenger who overcame a terrorist trying to highjack the flight, when others remained passive in their seats. Would Schuringa have done this, they ask, if he was averse to harming others?^20^

We should first note in passing that this worry is also based on a somewhat exaggerated reading of the empirical results. To start with, the only evidence we have so far about the effects of Citalopram relates to responses to hypothetical moral dilemmas which relate to situations where the harm is done to an innocent person, and where there is often no commonly agreed moral solution. By contrast, virtually everyone agrees that Schuringa did the right thing. He didn't face any kind of dilemma, and it's not as if the other plane passengers thought that it would be *wrong* to overcome the terrorist. They were simply afraid.^21^ It would thus be rash to claim that Citalopram would make people reluctant to harm others in situations where one needs to harm to prevent someone from harming others, and where nearly everyone agrees that this is the morally right thing to do.^22^

Perhaps more importantly, Harris and Chan seem to overlook the fact that the effect reported by Crockett et al. was driven by individuals who were high in empathic concern for others. Citalopram had no effect on the moral decisions of individuals who were low on such concern. It thus increases the aversion to harming only in a subset of individuals. For all we know, Schuringa was low on empathic concern, and wouldn't have been affected anyway.^23^

Finally, this largely anecdotal example is anyway a very weak argument. *Any* intervention has *some* negative consequences. The question is whether its effects are overall positive or negative. Harris and Chan do nothing at all to show that they would be negative.^24^

But let's set these worries aside. A more important lesson here is that, to the extent that levels of serotonin played any part in explaining why Schuringa acted as he did and others didn't (and for that matter, why the *terrorist* acted as he did and others didn't), then this must be due to significant individual variation in levels of serotonin (and thus of aversion to harming) within the human population.

That there is such variation is of course a simple empirical fact even if serotonin played no interesting part in what happened in this particular incident. Some people have more serotonin than others, and this contributes to affective and behavioural differences over a life time, including, presumably, differences in aversion to harming others. And the same goes for natural levels of testosterone, noradrenaline, oxytocin, dopamine, and so forth, all of which can have a profound influence on mood, cognition and other-directed attitude and behaviour.

The Harris and Chan argument implicitly presupposes that Schuringa had a lower level of serotonin than the more timid other passengers. There is no evidence that this is so but for our purposes here, we can accept this assumption.^25^ But if so, doesn't it follow that we should try to *reduce* the serotonin levels in the general population? We needn't aim to reduce everyone to Schuringa's purported level. Perhaps some diversity in aversion to harm is a good thing. But surely we can't just assume that, whenever a terrorist tries to highjack a plane, there will *always* be at least one passenger with Schuringa's serotonin level. This, too, is something we could try to actively address. So once again, an apparent objection to biomedical interventions targeting our moral capacities turns out to be, on closer inspection, a strong argument in their favour – and more specifically *in favour* of the pharmacological modulation of serotonin.

## Serotonin is Not an Alien Substance

From the way Harris writes about serotonin, one might get the impression that serotonin is some artificial substance that was given to the participants in the experiment. What participants were given was rather Citalopram, which is indeed a pharmaceutical product. But Citalopram works by blocking the re-uptake of serotonin, a neurotransmitter that of course naturally occurs in the brain, and is central to normal brain activity. From the way Harris writes, you might think it would be a good thing if we removed this bad influence. But if you were to remove all of the serotonin from someone's brain, the result will be disastrous.

This point is true of many current biomedical forms of cognitive or affective enhancement. They may involve external intervention in one's brain, but their *proximal effects* almost invariably operate by modulating naturally existing brain states and processes. It is thus highly misleading to write as if each of us starts in some pristine natural state, which biomedical interventions then must distort by introducing some entirely new process or substance.^26^

Given that all individuals already have a certain level of serotonin in their brain, the choice isn't between having or not having serotonin, but about what level of serotonin is best. And there are obviously three options: either the existing level is already optimal, or it would be better to raise it, or to decrease it.

This means that in order to oppose the biomedical modulation of serotonin, whether up or down, one must argue that the existing level is optimal.^27^

But why on Earth would anyone think that the existing level is optimal? Of course it can't be ruled out that it is. But this is a substantive empirical and normative claim which requires defense. And at least on the fact of it, it's *highly implausible* that our current level of serotonin is optimal. After all, our brains are products of evolution, which is a blind process that hardly seeks to maximize the good, or make us morally best. Evolution ‘cares’ only about reproductive success. Moreover, even if the evolutionary process somehow led to what is in one sense an optimal result, this result may be optimal only in the environment in which our very distant ancestors lived. It is very unlikely to be optimal in our utterly different modern environment. (There was, for example, no police in the primeval savannas, nor were there planes or hijackers …)

But if the current level isn't optimal, and we now have means of improving it (in whichever direction), then surely we have strong reasons to do so – including by biomedical means. Effective biomedical interventions that modulate serotonin are not merely farfetched speculations. They are already available, and widely used. Such interventions are not, of course, even remotely close to some kind of ‘good pill’ that would make us more virtuous. But this is exactly the point. Biomedical interventions that improve our moral decision-making needn't (and are unlikely to) take the form of such a magical virtue pill. They will instead simply modulate the various processes that are already operative in moral judgment and action.

To assume without argument that the current levels of some capacity or substance are optimal is a common mistake in the enhancement debate, which has already been diagnosed by Bostrom and Ord.^28^ For any property, X, we can ask: should we increase or enhance X? Opponents of enhancement typically argue that X should not be increased. But then we should ask the opposite question: should X be decreased? If *more* of X is *not* good, perhaps *less* of X is even better. Opponents of enhancement typically wish to resist this move – they typically believe we are at some local optimum for X where both increasing and decreasing it would be both be to the detriment to the organism. For example, *both* increasing *and* decreasing IQ, or empathy, or anxiety, or humour, would be detrimental. While there might be cases where evolution has created a local optimum for some function or capacity (like heart size), there will be many cases where this is not so.^29^ To ignore this possibility is a clear example of what psychologists call status quo bias. One of the aims of this article is to extend Bostrom and Ord's critique, and to identify some of the misconceptions about enhancement that drive this bias.

## General Lessons

We can now draw some general lessons. We saw that:

1. Biomedical enhancement typically works by modulating *existing* states or processes that underlie our various capacities.
2. The ‘natural’ level of these states or processes is set in substantial part by our biology, as selected by evolution, as well as by various environmental influences and social pressures.
  Since these influences are largely ‘blind’:
3. It is unlikely that this prior level is optimal, relative to our values.

Notice that this isn't the same as saying that the current level is simply random, and in *no way* geared to something useful or beneficial. The claim is just that it many cases, it would be significantly *better* if we set that level ourselves, as deemed best by explicit reflection on our values and the empirical facts, rather than leave it to be determined by ‘morally blind’ influences such as evolution or the genetic lottery. This claim would hold whether our values are utilitarian or deontological or something else entirely. It follows that:

(4) There is prima facie reason to either raise or lower that ‘natural’ level using (morally permissible) means, including biomedical ones.

To deny this conclusion, to insist without strong argument that things are best as they happen to be right now, would be an obvious instance of status quo bias.^30^

Chan and Harris complain, of serotonin, that ‘it impairs or short-circuits moral reasoning and induces us to act on the basis of emotion rather than rational consideration of the moral and social consequences of those actions.’^31^ Status quo bias may or may not involve emotion, but it's a nice example of a psychological force that ‘short-circuits moral reasoning’.

## The Problem of Normal Variation

Even if you reject (3), and argued, implausibly, that the current level *is* optimal or something close, you face another problem:

(5) In most cases, there *isn't* a single ‘natural’ level of a given human disposition or capacity. There is considerable natural variation. Some people have more, some less.^32^

This is very clear in the case of serotonin, as we have already seen.

Some variation in serotonin levels might be due to environmental influence. But it is certain to have a significant genetic component. Indeed, a recent study has identified genetic variations that affect serotonin production, and thus predict how people would respond to moral dilemmas.^33^ If there is some optimal level of serotonin, it is clear that not all of us have it naturally. Is it better, then, to let one's level of serotonin be decided by this genetic lottery, as opposed to a more direct, reasoned procedure? (Think of your own response to, say, the Footbridge dilemma we described earlier. Is it not disturbing to think that your inclination to judge that it's wrong (or right) to push a stranger to his death to save five others is in part determined by your genetics?)

We now know that differences in the level (or strength) of many other of our dispositions and capacities is similarly shaped in significant ways by differences in our genetic endowment. For example, a recent study reports that variations in a gene called the COMT gene, which is involved in the production of the neurotransmitter dopamine, could predict significant differences in the amount of money individuals donated to a charitable cause.^34^ And whatever one's substantive ethical views, or conception of ideal moral decision-making, it is hard to see what would justify leaving these individual differences to be determined by genetic chance. Even judgments of what constitutes a fair distribution of a fixed resource have significant genetic determinants.^35^

On top of the considerable variation between individuals, the levels of most substances and processes vary over time *within* individuals. We may have more or less serotonin at different points of the day, and in different contexts. And even these patterns can change as we age.

Now if there is some specific level, perhaps already currently present in some people, that *is* optimal, then it seems to follow that we should adjust the overall population levels to match that level. This is again an argument for a fairly radical form of moral enhancement.

To this it might be replied that it is not the level of any single person that is optimal, but the overall pattern in the entire population that is optimal. It is best to have some people with more serotonin, and some with less. There may be benefits to this natural diversity. Perhaps it is good to have a mixture of some people who are averse to harming others and others who are more aggressive.

But this reply faces the same set of objections we considered above. The current distribution of traits in the current population wasn't set to be best, in light of our values. It was shaped by processes that are largely blind and partly arbitrary. For example, it is far from obvious that there is any benefit in the existence of psychopaths, yet 1% of the population are psychopaths.^36^ But even if there was such a benefit, it would be absurd to hold that there is already a perfect balance of psychopaths to saints in our society.^37^

Even if it is good to have a certain diversity with respect to some feature – to have, for example, some people who are very averse to harming others and some who aren't – rather than some single optimal level throughout a population, it remains the case that the optimal pattern of that diversity is *itself* something that would be best decided by explicit deliberation, and often best implemented through direct intervention (biomedical or other) where possible. It shouldn't be decided by the partly random outcome of processes that are largely blind.^38^

## The Natural Level Isn't ‘Natural’

We spoke until now about the ‘natural’ level of substances such as serotonin, but of course that level is not itself the product of nature alone. Our environment has changed dramatically, and the currently normal levels are often profoundly influenced by our modern environment – current nutrition, hygiene and medicine have led to dramatic increases in longevity, height and even IQ, compared to our not so distant ancestors.

We have earlier argued that it's absurd to assume that evolution has selected exactly the optimal level of aversion to harm – even for the short and brutish life in the savanna, let alone for our modern environment. But in any event, our current levels of serotonin are not the products of evolution alone.

For example, there is considerable evidence that levels of serotonin are affected by our diet. Researchers have found that domesticated chickpea, as it was first cultivated in the ancient Middle East, has higher levels of tryptophan (a substance that the body uses to produced serotonin) compared to wild forms of chickpea. It has been argued that this is not an accident, but the result of a long selection process by ancient humans – these ancient humans of course knew nothing of tryptophan or serotonin, but may have noticed different effects of different types of chickpea, and groups that cultivated forms of chickpea that are higher on tryptophan may have enjoyed a competitive advantage.^39^

If this theory is correct, then ancient humans have *already* unwittingly engaged in what is in effect a form of affective and moral enhancement, leading to greater levels of subjective well-being, and greater levels of aversion to harming others. The current state of many humans is *already* enhanced, and it's hard to see a deep moral difference between dietary enhancement and pharmacological ones, except that the latter is more effective.

On a more speculative note, several researchers have suggested that violence and murder rates are higher in countries where the standard diet is based on corn, and thus contains less tryptophan.^40^ If this hypothesis is correct, this again vividly reminds us that our current levels of serotonin are set by a range of largely random and arbitrary factors – it is surely merely an accident that the diet of some cultures is based on maize rather than grain!

It is of course not only diet that affects serotonin levels in the human population. Millions of people are *already* using pharmacological means to modulate their serotonin levels. Citalopram isn't just a peculiar laboratory intervention. It is a widely used drug.^41^ Thus, although people don't currently take antidepressants like Citalopram in order to be morally better, it turns out that millions are *already* taking drugs that make them morally *different*, for better or worse.^42^ Should we stop using Citalopram, and antidepressants that work in similar ways, because of their supposedly adverse effects on moral decision-making? This sounds absurd, but whether or not it is absurd, the point remains that we can't avoid making explicit decisions about what levels of serotonin are best, at both the individual and population level.

## Mixed Effects, Side Effects, and Shifting Contexts

The argument developed in this article also offers an answer to the common objection that biomedical interventions have both positive and negative effects, that our brain and bodies are complex and interrelated systems so that for any intervention that seems to have a beneficial upshot, there will always be numerous negative and even harmful side effects.^43^ This is another recurring doubt that Harris has about biomedical interventions into moral decision-making:

I for one am sceptical that we would ever have available an intervention capable of targeting aversions to the wicked rather than the good. Of course if ever we do have the prospect of such precise and unequivocally good producing interventions, I will welcome them. But I remain doubtful and remain worried about the prospect of weakening possibly essential and essentially moral responses. This is a ‘baby and bathwater’ problem which may prove soluble; I hope it will, but fear it may be intractable.^44^

Applied to the case of serotonin, the worry is that ‘serotonin-induced ethics will not necessarily always reduce harm, may indeed in some circumstances increase harm.’^45^

This is of course true. But this is just to say that one's level of serotonin would have a multiplicity of effects, some positive, some perhaps negative. This, however, is not yet any kind of objection to modulating serotonin, since this causal complexity *is already present in the existing ‘state of nature’*. It is not introduced only by biomedical interventions. When we consider what would be better levels of serotonin, both at the individual and population level, we of course need to consider the whole range of effects, positive or negative. But this also means considering the balance of positive and negative effects of *current* levels of serotonin.^46^

Moreover, as pointed out above, that something has some negative consequences in some context is of course a very weak argument against using that thing. That's true of virtually anything. The real question is whether its consequences are positive or negative *all things considered*. Finding one example where the consequences of an intervention might be negative tells us almost nothing about this question.

Consider next another common objection to biomedical enhancement. It is sometimes objected that certain dispositions are beneficial in some contexts, but not beneficial, or even harmful, in others. In some contexts it's good to be averse to harm and violence, but in others such an aversion would be unhelpful or even fatal. The worry is that if we use biomedical means to set our levels of serotonin, or some other substance, we'll be stuck with a level that might be beneficial in some contexts but harmful in others.

This again is not at all an argument against enhancement but, rather, an argument for more precisely *fine-tuned* enhancement. In fact pharmaceutical interventions may allow us to modulate serotonin levels so that they are more *appropriate* to context (the school yard vs. the battlefield or hijacked airplane). Why settle for the serotonin level we happen to get through the genetic (and environmental) lottery, and hope that it somehow fits the often radically different decision contexts we face throughout our life time, when we can intelligently modulate it to fit these changing context? By contrast, relying on the normal variation in the population to provide a range of dispositions that might match different types of contexts – e.g. people who are less averse to harming others might make be good law-enforcers or soldiers – is a far cruder way of dealing with this problem. And again, even if in some cases it would be better (or practically unavoidable) to set fairly stable levels of some dispositions, it will remain the case that the degree and form of the variation within the population can itself be the object of reasoned choice rather than mere chance.

## Enhancing the Enhancement Debate

We have used Harris's remarks on Serotonin and moral enhancement to illustrate several common mistakes that are made in the enhancement debate. But these mistakes are by no means unique to Harris.

Michael Sandel, perhaps the most famous opponent of enhancement, argues that biomedical enhancement would compromise our openness to the unbidden, and reduce our sense of solidarity with others.^47^ Sandel ignores here both the fact that our current levels of openness to the unbidden and solidarity are shaped by partly arbitrary biological and social factors, and that these dispositions might themselves be subject to biomedical enhancement.^48^ Francis Fukuyama has similarly claimed that ‘[t]he first victim of transhumanism might be equality.’ This again ignores the point that one important driver of inequality is genetic and other biological differences between people. In fact, as one of us has argued, enhancement within the normal range may be *necessary* to achieve equality.^49^ And when Habermas worries that genetic selection would threaten autonomy,^50^ he forgets that the capacities that underlie autonomy also have a biological basis and exhibit considerable normal variability. These capacities are far from optimal in many individuals, and could themselves be targets for biomedical enhancement.^51^

In his critique of genetic enhancement, Michael Parker writes that:

In *All's Well that Ends Well*, Shakespeare has a minor character speak the following lines, ‘The web of our life is of mingled yarn, good and ill together; our virtues would be proud if our faults whipp'd them not, and our crimes would despair if they were not cherish'd by our virtues’ … Shakespeare is not simply reminding us that human lives are by their very nature characterised by both good and ill, and that we must learn to live with these aspects of ourselves and of those around us. He makes the stronger and ultimately more interesting claim that both strengths and weaknesses of character and of our lives more broadly, are essential and interdependent elements of the good life. Both aspects of our lives are interwoven and, indeed, it is this interweaving and our struggles with it that make us what we are and constitutes in its interplay of light and dark, much that is of value and significance in human existence.^52^

This echoes claims made by the President's Council in *Beyond Therapy*, and further propounded by Leon Kass, its President, on the value of suffering:

Traumatic memories, shame, and guilt, are, it is true, psychic pains. In extreme doses, they can be crippling. Yet, short of the extreme, they can also be helpful and fitting. They are appropriate responses to horror, disgraceful conduct, injustice, and sin, and, as such, help teach us to avoid them or fight against them in the future … there appears to be a connection between the possibility of feeling deep unhappiness and the prospects for achieving genuine happiness. If one cannot grieve, one has not truly loved. To be capable of aspiration, one must know and feel lack.^53^

Parker, Kass and the former President's Council all make the same mistake. Let us assume that the best life requires what Parker describes, after Shakespeare, as an interplay of light and dark. This no objection to enhancement, as one of us has previously argued.^54^ Some people have a lot of light and no dark; others are all dark. The issue is whether we should accept what nature delivers up or make a choice. Likewise for the claim that deep unhappiness being necessary for genuine happiness. It is highly unlikely that all people feel the right amount of shame, guilt and unhappiness in the situations Kass imagines.^55^

Whatever value we hold that makes for a good or virtuous life, that value will necessarily differ in different people, at different times. No individual or population can always instantiate that value to the optimum degree. So the door to enhancement, in the normal range, remains open.

## Conclusion

The aim of this paper was to draw attention to several common errors in thinking about enhancement. These errors are pervasive, and can influence even those who are generally enthusiastic about enhancement. Once these mistakes are made explicit, some familiar objections to enhancement lose much of their force. In fact, as we have shown, some apparent objections to enhancement turn out to actually be strong considerations in *favour* of enhancement, simply in a different direction.

The argument we developed here is neutral on whether we should be more utilitarian or more deontological, more or less emotional, or more or less averse to harm. We argue that, whatever one's view of the best set of dispositions, the current level of serotonin and similar biological properties is very unlikely to be optimal, and we have at least prima facie reasons to seek to modulate it using biomedical means. The same considerations would have similar force in the context of many other natural substances, capacities and dispositions which might be open to biomedical modulation.

In numerous discussions on enhancement, a recurring objection is that we do not know what is good, or what would constitute an improvement in human well-being or moral dispositions. This is, perhaps, the best diagnosis for the status quo bias that infects so many protagonists in the debate – since we don't know what would be better, we should remain where we are. We cannot fully address this large question here, though we have considered it elsewhere.^56^ It suffices to say that only radical relativist or nihilist could hold that there are no robust values that can guide enhancement. For example, one basic element of morality is willingness in certain situations to make self-sacrificial decisions for the benefit of others. Extreme pervasive and persistent selfishness is a vice. Yet many people are not prepared to make the self-sacrifice of people like Schuringa or indeed of a very limited kind necessary to deal with global problems like climate change or poverty.^57^ It is hardly controversial that some minimal level of altruism is desirable within any morality, deontological, utilitarian or other.

Human enhancement is sometimes understood to refer to radically transforming human capacities in ways that go beyond the normal range – what we have called supranormal human enhancement. Sometimes it is understood to refer merely to interventions that aim to improve human capacities in non-therapeutic ways, interventions that may still be well within the currently normal range. Much of the controversy has focused on the first, more radical sounding understanding of enhancement.^58^ But even changes that operate within the currently normal range can be dramatic. It would be dramatic enough if an intervention gave most people an IQ of 140, or a lifespan of 110, even if both figures are well within the normal range. And even interventions that just increase or reduce the current diversity of dispositions and capacities might, in some context, be very important. To see this, consider the point that the lifestyle common in Western countries is obviously within the current statistically normal variation. But it would nevertheless be an incredible achievement if most people on Earth could live like that. And even small effects can make a very significant difference when they are spread across a large population.

Dramatic forms of enhancement are more exciting, and for many, more frightening. These forms of enhancement naturally get the most attention. But they may not be feasible, and if they are feasible, they are not likely to be available in the near future. By contrast, we are already surrounded by more modest forms of biomedical enhancement, and others will almost certainly emerge in the near future. And even modest forms of normal range human enhancement, pursued on a wide scale, could have radical consequences.

## Footnotes

1 F. Fukuyama. 2002. *Our Posthuman Future: Consequences of the Biotechnology Revolution*. New York: Farrer, Straus & Giroux; J. Habermas. 2003. *The Future of Human Nature*. Cambridge: Polity Press; M. Sandel. 2007. *The Case Against Perfection: Ethics in the Age of Genetic Engineering*. Cambridge, MA: Belknap Press of Harvard University Press.

2 Frances Kamm notes both senses on enhancement in her ‘Is There a Problem with Enhancement?’ *Am J Bioeth* 2005; 5(3): 17–19.

3 These are actually worries that Harris shares with the bioconservative Michael Sandel who, in a 2007 interview, said that ‘… some people say, well suppose we could have some gene therapy that would enhance people's moral capacity … to imagine that those capacities could be deepened in a pill, would cause unimaginable damage to everything we understand about human moral character’. (*Tikkun*, September/October 2007). In personal communication, Harris clarifies that he is not opposed in principle to biomedical moral enhancement, but doubts that it will be desirable, all things considered.

4 J. Harris & S. Chan. Moral Behaviour is Not What it Seems, *Proc Natl Acad Sci U S A* 2010; 107(50): E183. In later papers, Harris offers rather idiosyncratic definitions of terms such as ‘moral enhancement’ and ‘moral judgment’ that diverge from their use by many others. In more recent articles, Harris clarifies that he is not opposed to moral enhancement in *his* sense, though he strongly suspects that it's highly unlikely, at best, that biomedical means can amount to such moral enhancement. For our purposes, we needn't engage with these issues, or with the other objections that Harris has to using biomedical means to modulate moral dispositions. Again, our primary aim here is not to defend moral enhancement, or any specific conception of moral enhancement, but to uncover some general errors in thinking about enhancement. To avoid these terminological issues, we shall therefore largely speak of biomedical interventions that modulate moral decision*-*making. Although we do not accept the way Harris defines ‘moral enhancement’, the arguments we develop have force *even* if one accepts this definition.

5 This experiment is reported in M. Crockett et al. Serotonin Selectively Influences Moral Judgment and Behaviour through Effects on Harm Aversion. *Proc Natl Acad Sci U S A* 2010; 107: 17433–17438. The Harris (and Chan) worries about serotonin, and biomedical moral enhancement in general, are raised in Harris & Chan *op. cit.* note 4; S. Chan & J. Harris. Moral Enhancement and Prosocial Behaviour. *J Med Ethics* 2011; 37: 130–131; J. Harris. Moral Enhancement and Freedom. *Bioethics* 2011; 25: 102–111. We focus on this study following Harris, but it only serves as an example of a more general point. There are, in any event, a wide range of other studies showing the effect on moral judgement of a range of other substances, including oxytocin, testosterone, and propranolol. See e.g. S. Terbeck et al. Beta-Adrenergic Blockade Reduces Utilitarian Judgment. *Biol Psychol* 2013; 92: 323–328; and for a review, Levy et al. Are You Morally Modified? The Moral Effects of Widely Used Pharmaceuticals. *Philos Psychiatry Psychol* forthcoming.

6 Harris & Chan *op. cit*. note 4.

7 Chan & Harris *op. cit.* note 5.

8 For relevant discussion, see G. Kahane & N. Shackel. Methodological Issues in the Neuroscience of Moral Judgment. *Mind Lang* 2010; 25: 561–582; G. Kahane et al. The Neural Basis of Intuitive and Counterintuitive Moral Judgement. *Soc Cogn Affect Neurosci* 2012; 7: 393–402.

9 Including by Crockett and her colleagues – see e.g. M.J. Crockett, L. Clark, T.W. Robbins. Reconciling the Role of Serotonin in Behavioral Inhibition and Aversion: Acute Tryptophan Depletion Abolishes Punishment-induced Inhibition in Humans. *J of Neurosci* 2009; 29(38): 11993–11999. Tryptophan is an essential precursor of serotonin. A significant lowering of tryptophan can be induced by drinking an amino acid mixture that lacks tryptophan and contains a large number of large neutral amino acids. This mixture reduces serotonin levels in the brain by stimulating protein synthesis, which utilizes tryptophan, as well as because the large amino acids ‘compete’ with tryptophan for the same transport system to the brain. Serotonin levels reach their lowest points several hours after this concoction has been drunk.

10 The baseline level of deontological response to the Footbridge case, for example, is around 90%. See F. Cushman, L. Young, M. Hauser. The Role of Conscious Reasoning and Intuition in Moral Judgment: Testing Three Principles of Harm. *Psychol Sci* 2006; 17: 1082–1089.

11 Harris may still think that it would be better to persuade people to give up these intuitions than to reduce their strength in this way. This is a large issue that goes beyond the scope of this article. It is enough to note here that utilitarians have so far made little progress along these lines, in part precisely *because* of the strength of opposing intuitions.

12 Anger, for example, increases utilitarian responses. See G. Ugazio et al. The Role of Emotions for Moral Judgments Depends on the Type of Emotion and Moral Scenario. *Emotion* 2102; 12: 579–590.

13 Harris, *op. cit.* Note 5.

14 There is considerable evidence that certain emotions play a key role in moral thought and behaviour (for rather strong statements of this view, see e.g. S. Nichols. 2004. *Sentimental Rules*. Oxford: Oxford University Press; J. Prinz. 2007. *The Emotional Construction of Morals*. Oxford: Oxford University Press), and indeed even for rational thinking in general (see e.g. A. Damasio. 1994. *Descartes' Error* New York: G.P. Putnam's Sons). Harris gives the impression that an ideal moral agent is one whose moral decision-making would be entirely insulated from emotion and intuition. But the actual people who most closely approximate this picture happen to be psychopaths … In fact, recent work has found that individuals higher on psychopathy also tend to make more utilitarian judgements in trolley dilemmas. See e.g. D.M. Bartels & D.A. Pizarro. The mismeasure of morals: Antisocial personality traits predict utilitarian responses to moral dilemmas. *Cogn* 2011; 121(1): 154–161.

15 J. Haidt. The Emotional Dog and its Rational Tail: a Social Intuitionist Approach to Moral Judgment. *Psychol Rev* 2001; 108: 814–834; J. Haidt. 2012. *The Righteous Mind: Why Good People Are Divided by Politics and Religion*. New York: Pantheon Books; J. Prinz, *op. cit*. note 14.

16 And if the worry is that the influence of serotonin (and emotional generally) on our moral dispositions is non-rational or even irrational, then again this worry must already forcefully apply to our current state.

17 In personal communication, Harris suggests he might be willing to endorse such biomedical enhancement in the ‘opposite’ direction.

18 These two worries are prominent in Sandel, *op. cit.* note 1.

19 Habermas, *op. cit.* note 1.

20 Cf. Harris & Chan, *op. cit*. note 4; Chan & Harris, *op. cit*. note 5, and elsewhere.

21 Harris notes some disanalogies, but still repeatedly presses this example.

22 There is however evidence tying lower serotonin with aggression. It's still not entirely clear whether people low on serotonin tend to be more aggressive because they are less averse to harming others, or whether, as seems more plausible, the causation is in the contrary direction.

23 You may think that Schuringa wouldn't have acted as he did if he was low on empathy. Even setting aside Schuringa's considerable self-interested motivation to overcome the terrorist, there is significant evidence that empathy is a far weaker source of moral motivation than, for example, anger. For discussion, see J. Prinz. Forthcoming. Is Empathy Necessary for Morality? In *Empathy: Philosophical and Psychological Perspectives*. A. Coplan & P. Goldie, eds. Oxford: Oxford University Press.

24 It is also worth noting that if we were able to make *everyone* averse to harming others – which is admittedly unrealistic – then there won't be a problem, since this would also include the terrorist that Schuringa disarmed. So these supposed bad effects of increasing aversion to harming thus apply only in contexts where this intervention only affects part of the population.

25 Though for all we know, Schuringa *himself* might have been taking Citalopram or similar antidepressant – these are in very wide circulation!

26 This is true of most of the forms of biomedical enhancement currently under discussion. Of course some conceivable forms of enhancement might involve the introduction of completely new processes and capacities – implanting computer chips in people's brains might be an example. In fact some existing external forms of enhancement, like computing and the internet, arguably *do* give people supranormal powers. These forms of enhancement are beyond the scope of this paper.

27 We can set aside here worries that assume that biomedical (as opposed to ‘natural’) means are in some way especially problematic, not only because it's hard to justify this normative distinction, but also because Harris himself rejects it. But as we shall show below, this distinction is anyway highly problematic in the context of serotonin.

28 See N. Bostrom & T. Ord. The Reversal Test: Eliminating Status Quo Bias in Applied Ethics. *Ethics* 2006; 116(4): 656–679.

29 See N. Bostrom & A. Sandberg. 2009. The Wisdom of Nature: an Evolutionary Heuristic for Human Enhancement. In *Human Enhancement*. J. Savulescu & N. Bostrom, eds. Oxford: Oxford University Press.

30 See again Bostrom & Ord, *op. cit.* note 29.

31 Chan & Harris, *op. cit.* note 5.

32 Indeed, even if (implausibly) evolution more or less tracked the optimal level of the disposition or capacity, natural variation means that some individuals, at the farthest reaches of the curve, end up with more or less than the optimal level.

33 A.A. Marsh et al. Serotonin Transporter Genotype (*5-HTTLPR*) Predicts Utilitarian Moral Judgments. *PLoS ONE* 2011; 6(10): e25148. Interestingly, there appear to be significant global variation in the prevalence of this gene, which is moreover associated with cultural differences in individualism vs. collectivism. See Chiao and Blizinsky. Culture-gene Coevolution of Individualism-collectivism and the Serotonin Transpotert Gene. *Proc R Soc B* 2010; 277: 529–537.

34 M. Reuter et al. Investigating the Genetic Basis of Altruism: the Role of the COMT Val158Met Polymorphism. *Soc Cogn Affect Neurosci* 2011; 6(5): 662–668.

35 B. Wallace et al. Heritability of Ultimatum Game Responder Behavior. *Proc Natl Acad Sci U S A* 2007; 104(40): 15631–15634.

36 As Persson and Savulescu point out in their book *Unfit for the Future* (2012. Oxford: Oxford University Press), in our technologically advanced modern world, even a single malevolent individual could potentially wreak immense damage. That it may have been useful to have a few psychopaths around in the hunter gatherer tribes of our distant ancestors is simply irrelevant.

37 It seems likely that there are more psychopaths out there than we could possibly need. But it's *more* plausible to think that we need *more* psychopaths than to that think that the *exact* current level is *optimal*.

38 We do not have space here to consider how such policies might be implemented. Let us just say that they needn't involve coercive measures of any kind – there are other ways to promote such outcomes.

39 Z. Kerem et al. Chickpea Domestication in the Neolithic Levant through the Nutritional Perspective. *J Archaeol Sci* 2007; 34: 1289–1293. It's of course not clear that Neolithic farmers selected these strains because they made people less aggressive and more averse to harm. Serotonin has other potentially beneficial effects, such as improving mood.

40 A.R. Mawson & K.W. Jacobs. Corn consumption, Tryptophan, and Cross-national Homicide Rates. *J Orthomolec Psychiatry* 1978; 7: 227–230; M. Voracek & U.S. Tran. Dietary Tryptophan Intake and Suicide rate in Industrialized Nations. *J Affect Disord* 2007; 98: 259–262. It is generally important to keep in mind that serotonin is influenced, and can be modulated by, not only by biomedical interventions, but also through diet, exercise and so forth. See S.N. Young. How to Increase Serotonin in the Human Brain without Drugs. *J Psychiatry Neurosci* 2007; 32(6): 394–399.

41 According to a 2005 report of the US Centers for Disease Control and Prevention, antidepressants are the most commonly prescribed type of drug in the USA. According to the FDA, in 2011 a total of approximately 31.5 million prescriptions were dispensed for citalopram from U.S. outpatient retail pharmacies, and approximately 7.2 million patients received a dispensed prescription for citalopram from U.S. outpatient retail pharmacies (see http://www.fda.gov/drugs/drugsafety/ucm297391.htm; accessed 20 July 2012). The number would be even higher if we include patients taking others antidepressants that modulate serotonin. And these are figures only for US patients.

42 See Levy et al., *op cit.* note 5.

43 For example, Quednow thinks that ‘[b]ecause of the vast complexity of the brain, it seems likely that we will not be able to overcome trade-offs between enhancement and simultaneous impairment by drugs.’ See B. Quednow. Ethics of Neuroenhancement: A Phantom Debate. *BioSocieties* 2010; 5: 153–156.

44 Harris, *op cit*. note 5.

45 Chan & Harris, *op. cit.* note 5.

46 It's also important to distinguish here the side effects of *Citalopram* (which on the whole appear to be relatively benign) and those of *serotonin* (which we all already ‘suffer’ from naturally).

47 Sandel, *op. cit.* note 1.

48 See G. Kahane. Mastery Without Mystery: Why there is no Promethean Sin in Enhancement. *J Appl Philos* 2011; 28(4): 355–368.

49 J. Savulescu. Justice, Fairness and Enhancement. *Ann N Y Acad Sci* 2006; 1093: 321–338.

50 Habermas. Op. Cit Note 1.

51 O. Schaefer, G. Kahane & J. Savulescu. Enhancing Autonomy. Under Review. We develop these responses to Sandel, Fukuyama and Habermas in great detail elsewhere. Here we only want to gesture at a common mistake underlying these different objections to biomedical enhancement.

52 M. Parker. The Best Possible Child. *J Med Ethics* 2007; 33: 279–283.

53 L.R. Kass et al. *Beyond therapy: biotechnology and the pursuit of happiness* Washington, DC, 2003: 298, 299. http://www.bioethics.gov/reports/beyondtherapy/beyond_therapy_final_webcorrected.pdf (accessed 5 Mar 2007).

54 J. Savulescu. In Defence of Procreative Beneficence: Response to Parker. *J Med Ethics* 2007; 33: 284–288.

55 See G. Kahane. 2011. Reasons to Feel, Reasons to Take Pills. In *Enhancing Human Capacities*. J. Savulescu, R. ter Meulen and G. Kahane, eds. Oxford: Wiley-Blackwell: 166–178.

56 J. Savulescu & G. Kahane. The Moral Obligation to Create Children with the Best Chance of the Best Life. *Bioethics* 2009; 23(5): 274–290.

57 See I. Persson & J. Savulescu. 2012. *Unfit for the Future: The Need for Moral Enhancement*. Oxford: Oxford University Press.

58 There is of course also plenty of debate about the actual use of more modest interventions, such as Ritalin or Prozac. But these are already used by millions of individuals, and generate far weaker opposition. These familiar interventions are also not usually the main concern of the grand general objections to enhancement at the centre of the current debate. In any event, the mistakes we have highlighted in this article are also pervasive in discussion of these more modest enhancers. Conversely, these are mistakes we should try to avoid even if more radical forms of enhancement *are* feasible. We are grateful to an anonymous referee for pressing us to clarify this point.

